# Molecular Dynamic Simulation of Defective Graphene Nanoribbons for Tension and Vibration

**DOI:** 10.3390/nano12142407

**Published:** 2022-07-14

**Authors:** Jia-Jia Mao, Shuang Liu, Lili Li, Jie Chen

**Affiliations:** 1 Beijing Key Laboratory of Nonlinear Vibrations and Strength of Mechanical Structures, Faculty of Materials and Manufacturing, Beijing University of Technology, Beijing 100124, China; jiajia.mao@bjut.edu.cn (J.-J.M.); liu201901127@emails.bjut.edu.cn (S.L.); jchen@bjut.edu.cn (J.C.); 2 School of Materials Science and Engineering, East China Jiaotong University, Nanchang 330013, China; 3 Architecture and Civil Engineering Research Center, Shenzhen Research Institute, City University of Hong Kong, Shenzhen 518057, China

**Keywords:** graphene, tension, vibration, vacancy, molecular dynamic simulations

## Abstract

As deformation and defects are inevitable during the manufacture and service of graphene resonators, comprehensive molecular dynamic (MD) simulations are performed to investigate the vibrational properties of the defective single-layer graphene sheets (SLGSs) during tension. Perfect SLGSs, SLGSs with single vacancy, SLGSs with low-concentration vacancies, and SLGSs with high-concentration vacancies are considered, respectively. The frequencies of the perfect and defective SLGSs at different stretching stages are investigated in detail. The effects of different external forces are also taken into account to study the vibration properties of the defective SLGSs. Results show that the perfect and defective SLGSs both successively perform four stages, i.e., the elastic stage, the yield stage, the hardening stage, and the fracture stage during stretching, and the elastic properties of the SLGSs are insensitive to the vacancy defects, while the ultimate strain is noticeably reduced by the vacancies. The single vacancy has no effect on the vibration properties of SLGS, while the frequency decreases with the increasing vacancy concentration for SLGS at the elastic stage. The frequency of yielded SLGS with a certain vacancy concentration is almost constant even with a varying external force.

## 1. Introduction

The two-dimensional (2D) nanomaterial with a monatomic layer, graphene, has attracted significant attention from the scientific and industrial communities since its first isolation in 2004. Due to its extremely high mechanical, electrical, optical, and thermal properties, graphene has been considered an ideal material for use in nanoelectromechanical systems (NEMS) [1,2,3,4,5,6], which are of great interest both for fundamental studies of mechanics at the nanoscale and for a variety of applications, including force [7], position [8], and mass [9] sensing. For practical use in NEMS devices and other applications, vibration behaviors are critical for the design and reliability of systems. Zhang et al. [10] investigated the vibrational frequency of rippled single-layered graphene sheets, providing insight for the next-generation nano-resonant device design. Many theoretical and numerical studies on the vibrations of the SLGS, double-layered and triple-layered graphene sheets related structures without any prestressing have been published [11,12,13,14]. Previous studies [1,2] suggested that random built-in tension is generated in the single-layer graphene sheets (SLGSs) resonators during the fabrication process. This paper focuses on the tensile behaviors and vibration properties of monolayer graphene resonators with pre-tension.

Similar to other known materials, perfect graphene sheets are almost impossible to manufacture, even when fabricating experimentally. It is quite well known that defects in materials have a great influence on the properties of crystals and nanostructures [15,16,17,18]. Defects in graphene are usually in the form of vacancies, dislocations, grain boundaries, etc., [19,20]. The static [21,22,23,24] and dynamic [25,26,27,28] mechanical properties of graphene sheets are sensitive to lattice imperfections. Fully understanding the tensile and vibration properties of defective graphene sheets is necessary for the reliability of graphene resonators. Due to the difficulties and uncertainties in nanoscale experiments, simulation methods are widely utilized as an alternative technique. Chu et al. [25] proposed the Monte Carlo-based finite element method to simulate the vibrations of graphene sheets with vacancies and reported a dramatic decrease in the foundational frequencies of the graphene sheets along with increasing vacancy defects. Several studies used classical molecular dynamic (MD) simulation studies to investigate the effect of varied defects on the tensile response of graphene and graphene reinforced composites [29,30].

In order to understand the vibrational properties of graphene nanoribbons at different tensile conditions, this paper treats the graphene nanoribbon as a finite-length pre-strained SLGS with clamped ends. The originality of the present work includes: (1) The tensile performances of SLGSs with different vacancy concentrations are studied, (2) the SLGSs with varying pre-strained degrees are taken into consideration to analyze their vibrational properties. We, first, perform extensive MD simulations to analyze the tensile characteristics of the finite-length SLGSs with different vacancy defects, and then study their natural frequencies with varying pre-strained degrees. The effect of different external loads on the resonant response of the pre-strained SLGS with clamped ends is also analyzed to ensure the safety of the graphene nanoribbons. All the calculations related to this work were done by the large-scale atomic/molecular massively parallel simulator (LAMMPS) an open-source molecular dynamic software on Windows (version; September 6, 2016).

## 2. Materials and Methods

**Figure 1:**
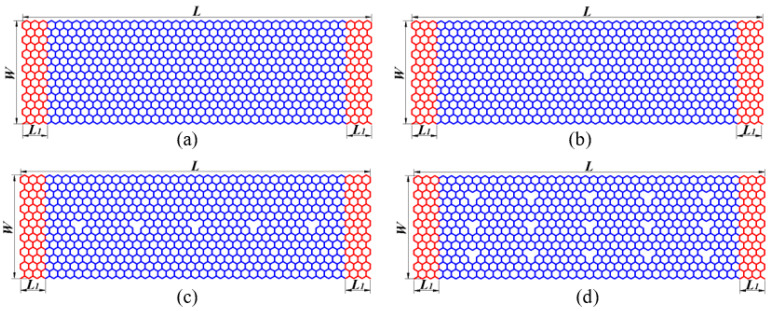
Atomic structures of (**a**) Perfect, (**b**) Single vacancy, (**c**) Low-concentration vacancies, and (**d**) High-concentration vacancies graphene nanoribbons.

Figure 1 shows the atomic structures of defective graphene nanoribbons with length *L* = 10.365 nm, thickness *h* = 0.335 nm, and width *W* = 3.24 nm, including (a) perfect sheet, (b) single vacancy, (c) low-concentration vacancies, and (d) high-concentration vacancies SLGSs. Three layers at both ends, colored in red, of all the SLGSs are fixed, while the rest are free parts that are colored in blue. A periodic boundary is used in the X and Y directions, and a shrink-wrapped boundary is used in the Z direction.

In the MD simulations, the adaptive intermolecular reactive empirical bond order potential (AIREBO) is adopted. As given in detail in Stuart et al. [31], the AIREBO potential can be represented by a sum over pairwise interaction:

(1) $$E=\frac{1}{2}\sum_{i}\sum_{j\neq i}\left[E_{ij}^{\mathrm{REBO}}+E_{ij}^{\mathrm{LJ}}+\sum_{k\neq i,j}\sum_{l\neq i,j,k}E_{ijkl}^{\mathrm{TORS}}\right]$$

where $E_{ij}^{\mathrm{REBO}}$, $E_{ij}^{\mathrm{LJ}}$, and $E_{ijkl}^{\mathrm{TORS}}$ are respectively the covalent bonding REBO interactions, LJ terms, and torsion interactions [31].

Each model is initially relaxed using canonical ensemble (NVT) simulation until the energy of the system is fully minimized for a specified temperature of 100 K, then axial strain applied. The deformation-control method with a strain rate of 0.0005 ps^−1^ is implemented along the positive X-axial until the graphene nanoribbons collapse. The strain increment is applied after every 1000-time steps. The Velocity–Verlet algorithm and Nosé–Hoover thermostat are used.

## 3. Results and Discussion

**Figure 2:**
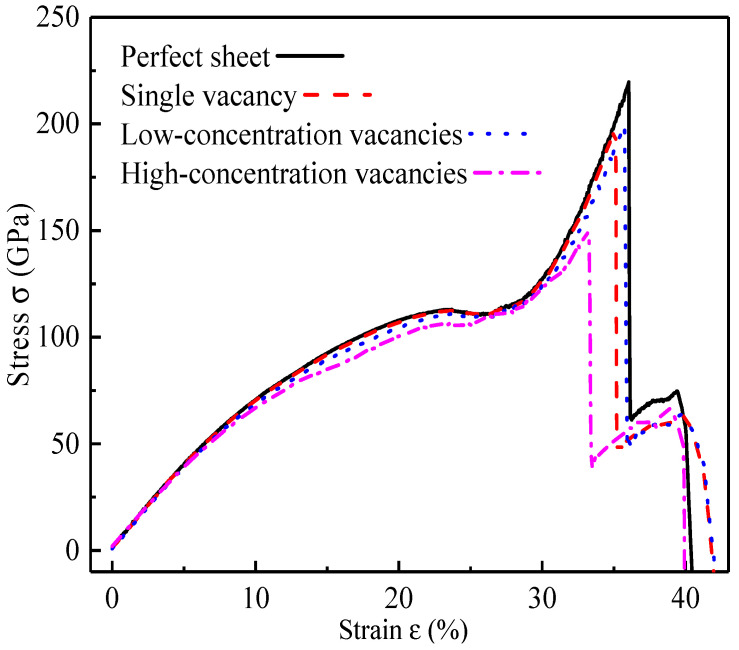
Tensile stress-strain curves of SLGSs with different vacancy concentrations.

After computing and exporting the stress and geometrical characteristics of the defective SLGSs during tension through the LAMMPS codes, the tensile stress-strain curves of the SLGSs with different vacancy concentrations are obtained, as shown in Figure 2. In this work, the SLGSs with the same vacancy concentrations but different vacancy locations are also calculated. It should be pointed out that they have the same performance. Results show that the tensile processes of the SLGSs go through four stages, namely, the elastic stage, the yield stage, the hardening stage, and fracture [32]. Obviously, linear and nonlinear elastic processes are both observed. Interestingly, the linear elastic behaviors and the yield strains of these defective SLGSs are similar, but their nonlinear elastic performances are different. Besides, the perfect sheet and the SLGS with a single vacancy almost have the same elastic phenomena, while a notable gap is observed between their hardening stages. It implies that vacancies make a significant difference in the ultimate strain of SLGSs, even a single vacancy.

**Figure 3:**
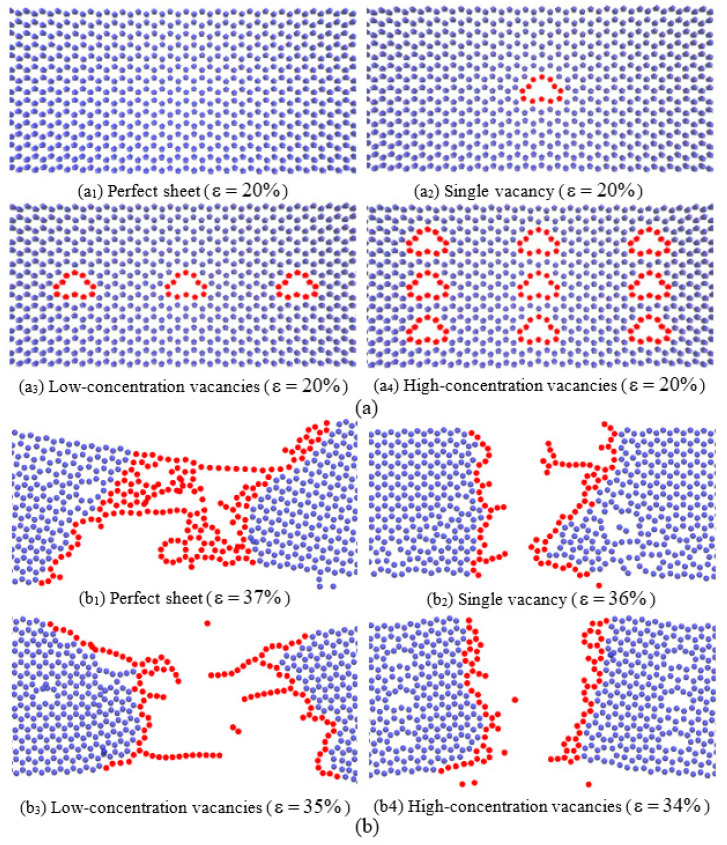
Local atomic configurations of SLGSs with different vacancy concentrations during (**a**) elastic (ε = 20%), (**a_1_**) Perfect sheet, (**a_2_**) Single vacancy, (**a_3_**) Low-concentration vacancies and (**a_4_**) High-concentration vacancies, and (**b**) fracture processes, (**b_1_**) Perfect sheet, (**b_2_**) Single vacancy, (**b_3_**) Low-concentration vacancies and (**b_4_**) High-concentration vacancies.

To explain the vacancy sensibility, Figure 3 shows the atomic configurations of the SLGSs with different vacancy concentrations during (a) elastic (ε = 20%) and (b) fracture processes. During the elastic stage, overall stretches are observed for all the SLGSs. Local irregularities appear around the vacancies for the defective SLGSs. As the proportion of the local irregularities for the single vacancy SLGS can be negligible, the SLGS with a single vacancy has the same elastic properties as the perfect one, as shown in Figure 2. However, their failure mechanisms are totally different in our calculations. Specifically, the defective SLGSs collapse with perfect clear-cut configurations while the perfect SLGS fracture with a relatively rough fracture surface [33].

In order to investigate the vibrational features of the defective SLGSs at different stretching stages, different pre-strains are first subjected to the system as part of the process in our tensile experiments. Three SLGSs pre-strained at 10%, 20%, and 25% are studied, which respectively stand for the linear elastic stage, nonlinear elastic stage, and fracture. Seven different external forces *f* (17.04 nN, 25.26 nN, 34.08 nN, 42.6 nN, 51.12 nN, 59.64 nN, and 68.16 nN) per atom (blue in Figure 2) are respectively applied to the pre-strained SLGS perpendicularly to study the resonant responses of the pre-strained SLGSs in simulations. The external load is removed after 10,000-time steps of loading. When the energy of the system is minimized again, the NVT simulation turns to the micro-canonical ensemble (NVE) simultaneously in each simulation to ensure the conservation of energy during vibration. Because of the C-C bonds and the energy contributed by an external force, the systems begin to oscillate, which is accompanied by the conversion between kinetic energy and the potential energy of the systems.

**Figure 4:**
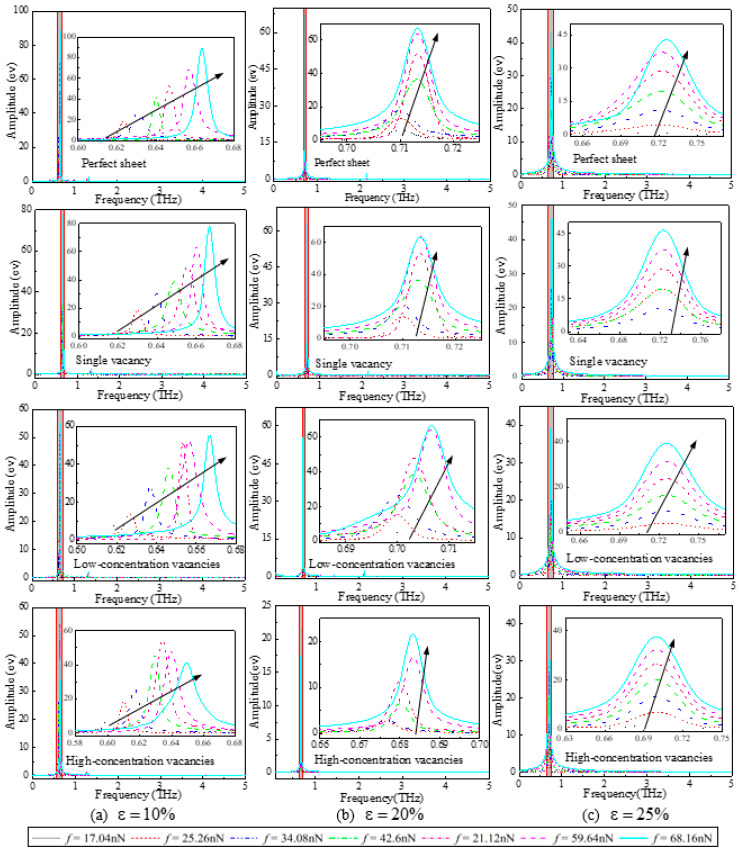
Frequency response curves of the SLGSs with different vacancy concentrations under (**a**)ε = 10%, (**b**) ε = 20%, and (**c**) ε = 25% pre-strained (the trend arrows represent the increasing external forces *f*).

Considering the period of the conversion between kinetic energy and potential energy as T, the vibrational period should be 2T. Frequency response curves can be obtained through Fast Fournier Transform (FFT) by selecting the kinetic energy of the systems as time-domain signals. Figure 4 gives the frequency response curves of the SLGSs with different vacancy concentrations under (a) ε = 10%, (b) ε = 20%, and (c) ε = 25% pre-strained conditions. For all these different kinds of defective SLGSs, the external force influences the frequency responses of SLGSs with 10% pre-strained the most and affects the frequency responses of SLGSs with 25% pre-strained the least. In other words, the vibration properties of SLGSs during the elastic stage are the most sensitive to the external load. It is noted that the frequencies shown in Figure 4 are twice the resonance frequencies of corresponding systems.

**Figure 5:**
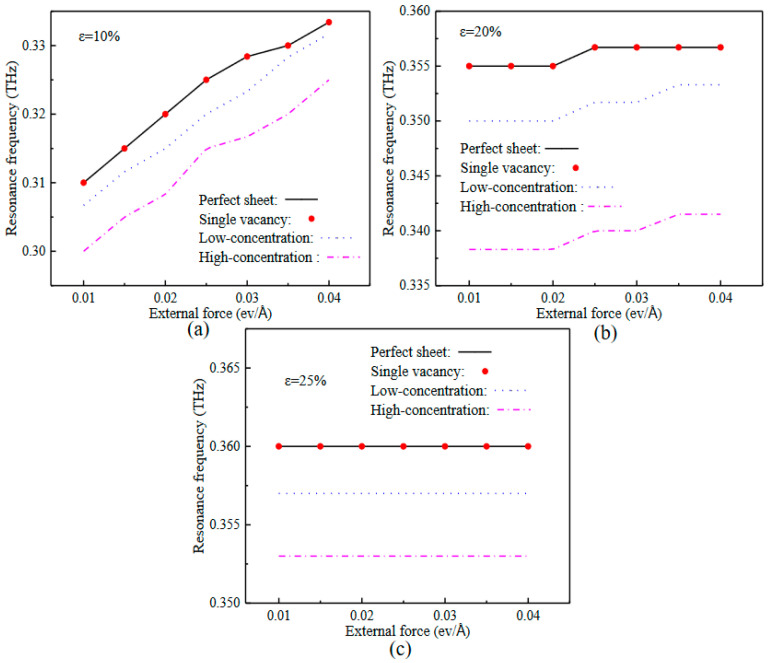
Relationship between the external forces and resonance frequencies of the defective SLGSs at (**a**) linear elastic stage (ε = 10%), (**b**) nonlinear elastic stage (ε = 20%), and (**c**) yield stage (ε = 25%).

Figure 5 illustrates the relationship between the external force and the resonance frequencies of the defective SLGSs at (a) linear elastic stage (ε = 10%), (b) nonlinear elastic stage (ε = 20%), and (c) yield stage (ε = 25%). As seen, the resonance frequencies of the SLGSs with a single vacancy are almost the same as that of the perfect sheets, and more vacancies lead to a lower resonance frequency for a certain external force because of the reducing stiffness. In Figure 5a, the defective SLGSs with 10% pre-strain, i.e., at the linear elastic stage, the resonance frequencies almost linearly increase with the increasing external force. There is a notable platform with varying external force for the defective SLGSs at nonlinear elastic stage (ε = 20%), Figure 5b. In Figure 5c, the resonance frequencies of the SLGSs with a certain vacancy concentration, nearly stabilize at a constant value, which is independent of the external force. Such different phenomena for the SLGSs at different stretching states are caused by the elastic potential energies transformed from the work done by the external forces. For the SLGSs stretching closer to the yield strain, less elastic deformation can be afforded, which leads to less sensitive vibration properties to the varying external load.

## 4. Conclusions

In summary, extensive MD simulations are performed to understand the tensile strength of finite-length graphene nanoribbons with varying vacancy concentrations and their vibration properties at different stretching states. The graphene nanoribbon is simulated by SLGS. Perfect SLGSs, SLGSs with a single vacancy, SLSGs with low-concentration vacancies, and SLSGs with high-concentration vacancies were considered. Through the tensile stress-strain curves, all the SLGSs with different vacancy concentrations went through the four stages, i.e., elastic stage, yield stage, hardening stage, and fracture during the tensile process. The vacancies have a greater influence on the ultimate strain of the SLGSs, than the elastic limit and yield limit. As for the vibrations of the defective SLGSs, different external forces are respectively applied to investigate their resonance frequencies at different stretching stages. Even the single vacancy had no effect on the vibrational properties of the SLGSs, the increasing vacancy concentration decreases the resonance frequencies of the SLGSs at the elastic stage. The frequency of yielded SLGS with a certain vacancy concentration is almost constant even with a varying external force.

## Data Availability

Data are available from the authors upon reasonable request.
